# Changes in urine composition after trauma facilitate bacterial growth

**DOI:** 10.1186/1471-2334-12-330

**Published:** 2012-11-29

**Authors:** Cecile Aubron, Olivier Huet, Sylvie Ricome, Didier Borderie, Eric Pussard, Pierre-Etienne Leblanc, Odile Bouvet, Eric Vicaut, Erick Denamur, Jacques Duranteau

**Affiliations:** 1 Site Xavier Bichat, INSERM U722 and Université Paris 7 Denis Diderot, 16 rue Henri Huchard, Paris 75018, France; 2 Hôpital de Bicêtre, Département d’Anesthésie Réanimation, Assistance Publique - Hôpitaux de Paris and Université Paris 11 Le Kremlin Bicêtre, Paris, France; 3 Hôpital Cochin, Laboratoire de Biochimie, Assistance Publique - Hôpitaux de Paris and Université Paris 5, Paris, France; 4 Service de Génétique Moléculaire, Pharmacogénétique et Hormonologie, Hôpital de Bicêtre, Assistance Publique - Hôpitaux de Paris and Université Paris 11 Le Kremlin Bicêtre, Paris, France; 5 Faculté de Médecine, Site Villemin, URC and Université Paris 7 Denis Diderot, Paris, France

**Keywords:** Nosocomial urinary tract infection, *Escherichia coli*, Bacterial growth, Trauma patients

## Abstract

**Background:**

Critically ill patients including trauma patients are at high risk of urinary tract infection (UTI). The composition of urine in trauma patients may be modified due to inflammation, systemic stress, rhabdomyolysis, life support treatment and/or urinary catheter insertion.

**Methods:**

Prospective, single-centre, observational study conducted in patients with severe trauma and without a history of UTIs or recent antibiotic treatment. The 24-hour urine samples were collected on the first and the fifth days and the growth of *Escherichia coli* in urine from patients and healthy volunteers was compared. Biochemical and hormonal modifications in urine that could potentially influence bacterial growth were explored.

**Results:**

Growth of *E. coli* in urine from trauma patients was significantly higher on days 1 and 5 than in urine of healthy volunteers. Several significant modifications of urine composition could explain these findings. On days 1 and 5, trauma patients had an increase in glycosuria, in urine iron concentration, and in the concentrations of several amino acids compared to healthy volunteers. On day 1, the urinary osmotic pressure was significantly lower than for healthy volunteers.

**Conclusion:**

We showed that urine of trauma patients facilitated growth of *E. coli* when compared to urine from healthy volunteers. This effect was present in the first 24 hours and until at least the fifth day after trauma. This phenomenon may be involved in the pathophysiology of UTIs in trauma patients. Further studies are required to define the exact causes of such modifications.

## Background

Urinary tract infections (UTIs) are the most common infections acquired by hospitalized adult patients, with an estimated prevalence as high as 10%. UTIs account for up to 40% of hospital-acquired infections [1,2]. In approximately 15% of cases of nosocomial bacteraemia, the urinary tract is implicated as the portal of entry [1]. Several studies have reported that UTIs increase morbidity, healthcare cost and length of hospital stay [3-5]. Asymptomatic bacteriuria is a common cause of inappropriate antibiotic prescription, which may play a role in the emergence of resistant pathogens. Therefore, it is important to reduce the rate of hospital-acquired UTIs.

The occurrence of UTIs is increased in the presence of indwelling urinary catheters, particularly over time. Urinary catheterisation is a major identified risk factor of urosepsis because its presence allows bacteria to colonise the urinary tract [6]. The other identified independent risk factors for bacteriuria in the intensive care unit (ICU) are gender (female), length of ICU stay, and severity score at admission [7]. Many of the pathological states of critically ill patients lead to relative immunosuppression, with impairment of the ability of neutrophils to kill bacteria [8]. The ability of urine from seriously ill patients to support bacterial growth may also play an important role in the occurrence of UTIs. But, the role of urine composition in bacterial proliferation in critically ill patients has not been studied.

In normal individuals, urine is limited in nutrients, which are essential to bacterial growth, such as iron [9]. Urinary amino acids are important sources of carbon, nitrogen and sulphur, and play a key role in bacterial defences against osmotic stress [9-11]. Urine composition in trauma patients can be modified by inflammation, systemic stress, rhabdomyolysis, the treatment received for life support and possibly by the presence of a urinary catheter. For example, endogenous or exogenous catecholamines in urine could promote bacterial growth and facilitate bacteriuria, leading to an increase in susceptibility to infection [12].

The aim of this study was to determine if urine of trauma patients facilitates bacterial proliferation compared to urine of healthy volunteers and to describe urine composition changes, which may promote bacterial growth.

## Methods

### Patients

Adult trauma patients admitted to the 22-bed surgical ICU from the university hospital of Bicêtre (AP-HP, France) with an Injury Severity Score (ISS) [13] higher than 16 and requiring an indwelling urethral catheter were eligible for enrolment. Patients were not included if (i) antibiotic therapy was predicted to be necessary for clinical care when admitted to the ICU (except for prophylactic antibiotics), (ii) hospitalization occurred before ICU admission, (iii) the expected duration of stay in the ICU was shorter than 5 days, (iv) they were less than 18 years of age or pregnant, or (v) they had genitourinary trauma. The ethics committee of the Lariboisière Hospital (Paris, France) agreed to the protocol used in this research. The informed written consent was deemed non applicable as patient samples were taken as part of standard care.

The demographic characteristics of each patient, including age and gender, were recorded. The severity of illness was assessed by Simplified Acute Physiology Score (SAPS) II [14], Systemic Organ Failure Assessment (SOFA) score [15], and ISS at admission to the ICU, and (for the SOFA score) at five days. The following clinical and laboratory variables were recorded at the time of urine collection, at the first and the fifth days of ICU hospitalisation: (i) interventions including antibiotic administration from the first to the fifth day (antibiotic used, dose and duration which allowed us to determine whether antibiotics were given for prophylaxis or treatment), invasive mechanical ventilation and catecholamine infusion; (ii) biochemical data including arterial lactate (normal values: 0.5 to 2.2 mmol/L), plasma pH (7.40 ± 0.2), 24-hour urine flow, serum creatinine (normal values: 60 to 120 μmol/L), creatinine clearance (normal values > 80 mL/min), blood urea (normal values: 4 to 7 mmol/L), plasma creatinine kinase level (normal values < 250 U/L) ; (iii) and urinary bacteriology.

### Urine samples

Sterile urine samples over 24 hours were collected with an indwelling urinary catheter at day 1 (D1) and day 5 (D5) of the stay in the ICU. After centrifugation and filtration (0.22 μm pore size), urine samples were stored at −20°C.

Sterile urine from 6 healthy male volunteers (HV) aged between 22 and 50 years old [median of 28 years, (IQR, 25.5, 32.5)] with no history of UTIs or antibiotic use during the previous two months was collected. Furthermore, HV were free of iron intake. Before being stored at 4°C if used in the following 48 hours, or at – 20°C, midstream urine from at least three of the six HV were pooled. Twelve urine batches were studied.

### Urinary biochemical assays

The volume, biochemistry sodium (normal values: 50 to 200 mmol/24 hours), potassium (normal values: 50 to 150 mmol/24 hours), urea (normal values: 200 to 500 mmol/24 hours) and creatinine (normal values: 9 to 16 mmol/24 hours), pH (normal values: 5 to 7), osmolality (normal values: 700 to 1000 mosm/L, glucose, protein (normal values < 0.1 g/24 hours) and urinary sediment were recorded for each urine sample. Urinary glucose concentration higher than 0 mmol/l was considered as abnormal, based on the pathology lab‘s definition. The concentration of free iron was measured by spectrophotometry on a Modular PP analyser (Fer/Roche®; Roche Diagnostic) in urine of patients and HV. Urine samples were firstly acidified to a pH <2 to separate iron from carrier proteins. As described previously [16], ascorbate was added to allow the reduction of Fe^3+^. Then, FerroZine was used to determine the free iron concentration.

The urinary concentrations of nitrates were measured as described previously [17]. The levels of amino acids were determined using High Performance Level Chromatography (HPLC) on 24 samples (9 from urine of HV, 15 from 8 patients (8 samples collected on day 1, and 7 on day 5) [18]. The concentrations of norepinephrine, dopamine and epinephrine, which are known to influence bacterial growth, were measured on 24 samples from patients (12 samples collected on day 1 and 12 collected on day 5). Catecholamines were extracted from urine by ion exchange chromatography followed by alumina adsorption. Extracted catecholamines were separated and quantified by an HPLC method with amperometric detection [19,20]. This method is specific for catecholamines and does not detect oxidized derivatives such as o-quinone or adrenochrome. The concentrations of urinary catecholamines of trauma patients were compared with reference values measured in a healthy population (dopamine < 630 μg/g of creatinine, epinephrine <16 μg/g of creatinine and norepinephrine < 77μg/g of creatinine) [19].

### Bacterial strains

Two strains of *E. coli* were studied. *E. coli* CFT073, the prototype of the uropathogenic (UPEC) strains, was originally isolated from the blood and urine of a woman admitted to the University of Maryland Medical System (USA) [21]. *E. coli* CFT073 is fully sensitive to antibiotics. The second strain is *E. coli* R1B8J20 isolated during a septicaemia recorded in the prospective COLIBAFI study [22]. *E. coli* R1B8J20 is resistant to beta-lactams due to over-production of AmpC cephalosporinase. The minimal inhibitory concentrations for the following antibiotics, determined using an E-test method, were: ceftazidime >256 mg/L, cefoxitin >192 mg/L, cefepime = 16 mg/L, cefotaxime = 12 mg/L, amoxicillin-clavulanic acid >256 mg/L. This strain was also resistant to quinolones, kanamycin, streptomycin and sulfonamides. *E. coli* R1B8J20 allowed an evaluation of bacterial growth in urine of patients who had received antibiotics.

### Bacterial growth assays

Both *E. coli* strains were cultured in 4 ml of Luria Bertani medium for 18 hours at 37°C. The overnight cultures were centrifuged (4500 rpm during 15 min) and washed with 4 ml of isotonic saline serum. Forty ml of urine were inoculated with the volume of the overnight culture required to obtain a starting optical density of 0.01 at 600 nm (OD_600_), which corresponded to 2×10^6^ bacteria/mL, and incubated at 37°C with an agitation at 180 rpm. Optical density was measured at intervals with an Ultrospec 1100 pro (Pharmacia^®^) spectrophotometer, until the culture reached stationary phase or at least over 420 minutes.

Experiments were normally carried out using *E. coli* CFT073. When the patient received prophylactic antibiotics or therapeutic antibiotic therapy before the fifth day that was not predicted at admission, the resistant *E. coli* R1B8J20 isolate was also studied. Experiments were done in replicates.

To study the role of iron in bacterial proliferation, *E. coli* CFT073 growth in urine from trauma patients was studied after addition of an iron chelator, desferrioxamine (0.1 mM), using three urine samples collected on the first day from patients who were not on antibiotics. The inhibitory effect of desferrioxamine was expressed by percentage of growth inhibition after 360 minutes of growth.

The effect of norepinephrine was studied after addition of 1 μM, 10 μM or 50 μM to HV urine samples, using the method described by Freestone et al. [23]. These experiments were carried out using two independent HV urine samples.

### Statistical Analysis

The clinical and biological characteristics recorded on days 1 and 5 were compared using the Student *t* test (MedCalc). The biochemical characteristics of urine from trauma patients on days 1 and 5 were compared with the biochemical characteristics of urine from HV. When statistical distribution was Gaussian, mean difference in means and 95% confidence interval (CI) are shown. In cases of non Gaussian statistical distribution, the median difference and non parametric Hodges-Lehman 95% CI are shown. The growth of *E. coli* CFT073 in urine from patients without antibiotics on day 1 was compared with *E. coli* CFT073 growth in HV urine, using an analysis of variance for repeated measures (Statview). The same analysis was carried out for urine collected on day 5, and for the resistant strain *E. coli* R1B8J20. Finally, growth in urines from the first and the fifth days was compared. Spearman’s nonparametric correlation coefficient was used to correlate clinical or biological parameters and growth (Statview). The results are expressed in mean ± standard deviations. Statistical significance was fixed at 0.05 (two-tailed).

## Results

### Patients and urine samples

Twenty-two patients with severe trauma were recruited to the study over a period of 11 months.

**Figure 1:**
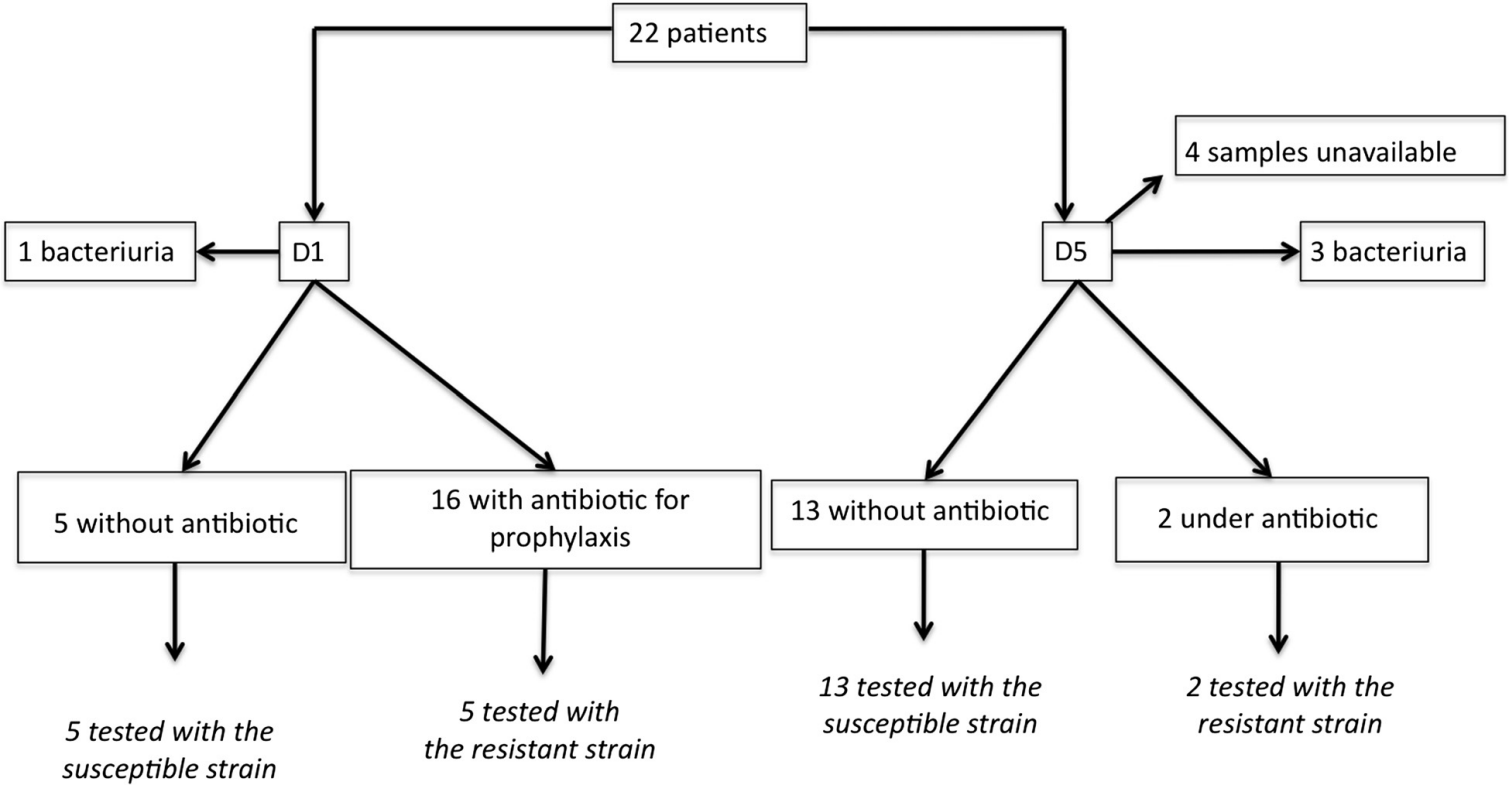
**Study design. **Twenty-two patients were included. Urine was collected on the first and fifth day after trauma to evaluate bacterial growth.

Of these 22 patients, sixteen received prophylactic antibiotics on day 1, and one had a positive bacteriological urine culture (*Proteus mirabilis* 10^5^/mL) (Figure 1). On day 5, two patients had received antibiotics from day 2 for a hospital-acquired infection, three patients had positive bacteriological samples (>10^5^/mL of *P. mirabilis*, coagulase-negative *Staphylococcus*, *Enterococcus faecalis*) and 4 of 22 samples were not available because the indwelling catheter was removed before day 5 (Figure 1).

The demographic and clinical characteristics are summarized in Table 1. On day 5, the majority of patients showed signs of recovery, demonstrated by a decrease in the SOFA score and the number of patients requiring life support. The significant improvement in biological parameters such as the creatinine clearance and the serum lactate level also demonstrated this trend (Table 2).

**Table 1:** Demographic and clinical characteristics of the 22 trauma patients on day 1 and on day 5 after the onset of trauma

**Variables**		**D1**	**D5**
Ages, years (mean ± SD)	35 ± 14		
Male, n (%)	15 (68%)		
ISS (mean ± SD)		30 ± 12	
SAPS II (mean ± SD)		33 ± 16	
SOFA score (mean ± SD)		6 ± 3	3 ± 4
Patients with invasive MV, n (%)		16 (73%)	11 (50%)
Patients with catecholamines, n (%)		16 (73%)	5 (23%)
Patients with antibiotics, n (%)		16 (73%)	2 (9%)
Urine output, mL/24h (mean ± SD)		1800 ± 890	2500 ± 1000

*MV *Mechanical ventilation, *ISS *Injury Severity Score, *SAPS II* Simplified Acute Physiology Score, *SOFA *Systemic Organ Failure Assessment, *D1 *on day 1, *D5 *on day 5.

**Table 2:** Biological characteristics of the 22 trauma patients on day 1 and on day 5 after the onset of trauma

**Variables**	**D1**	**D5**	***P***
Plasma pH	7.32 ± 0.07	7.40 ± 0.05	0.0001
Lactatemia (mmol/L)	1.9 ± 1.3	1.0 ± 0.4	0.005
Creatinine clearance (mL/min)	112 ± 55	191 ± 101	0.0025
Uremia (mmol/L)	4.9 ± 1.8	4.9 ± 2.3	ns
Creatininaemia (μmol/L)	76 ± 36	58 ± 20	0.0466
Bacteriuria, n (%)	1 (4.5%)	3 (14%)	ns

*D1 *on day 1, *D5 *on day 5.

### Bacterial growth assays

**Figure 2:**
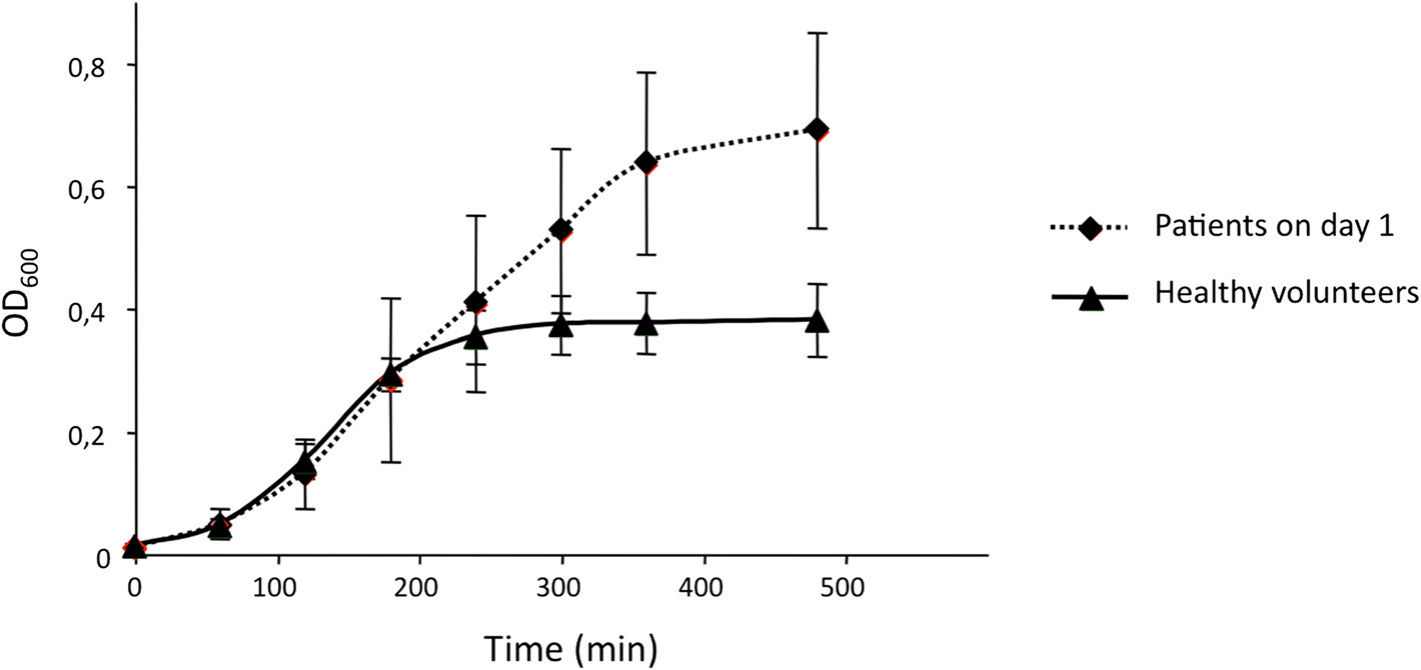
**Comparison of the growth of *E. coli *CFT073 in urine, collected on day 1, from trauma patients not receiving antibiotic (n = 5) (dashed line), and from healthy volunteers (n = 12) (complete line) (*p *= 0.0028).**

**Figure 3:**
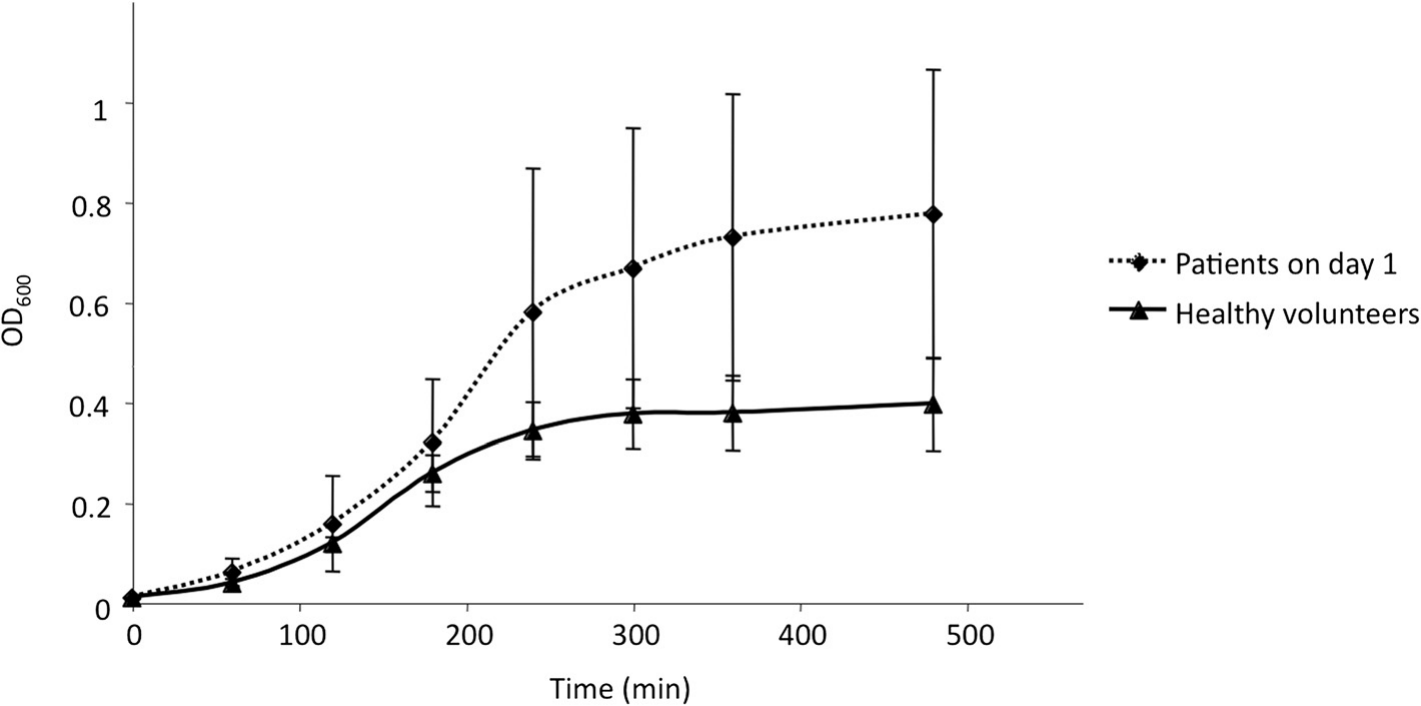
**Comparison of the growth of *E. coli *R1B8J20 in urine, collected on day 1, from trauma patients receiving prophylactic antibiotics (n = 4) (dashed line), and from healthy volunteers (n = 7) (complete line) (*p *= 0.0371).**

On day 1, for urine from five patients who were not treated with an antibiotic, the growth of the susceptible strain, *E. coli* CFT073, was statistically higher than in HV urine (*p* = 0.0028) (Figure 2). For the 16 patients who received prophylactic antibiotics on day 1, the growth of *E. coli* R1B8J20, the resistant strain, was studied in 5 urine samples. Growth was inhibited in 1 sample. The 4 remaining urine samples supported a significantly better growth than HV urine (*p* = 0.0371) (Figure 3). When tested in HV urine or in TP urine, there was no difference in growth between the two strains (data not shown).

**Figure 4:**
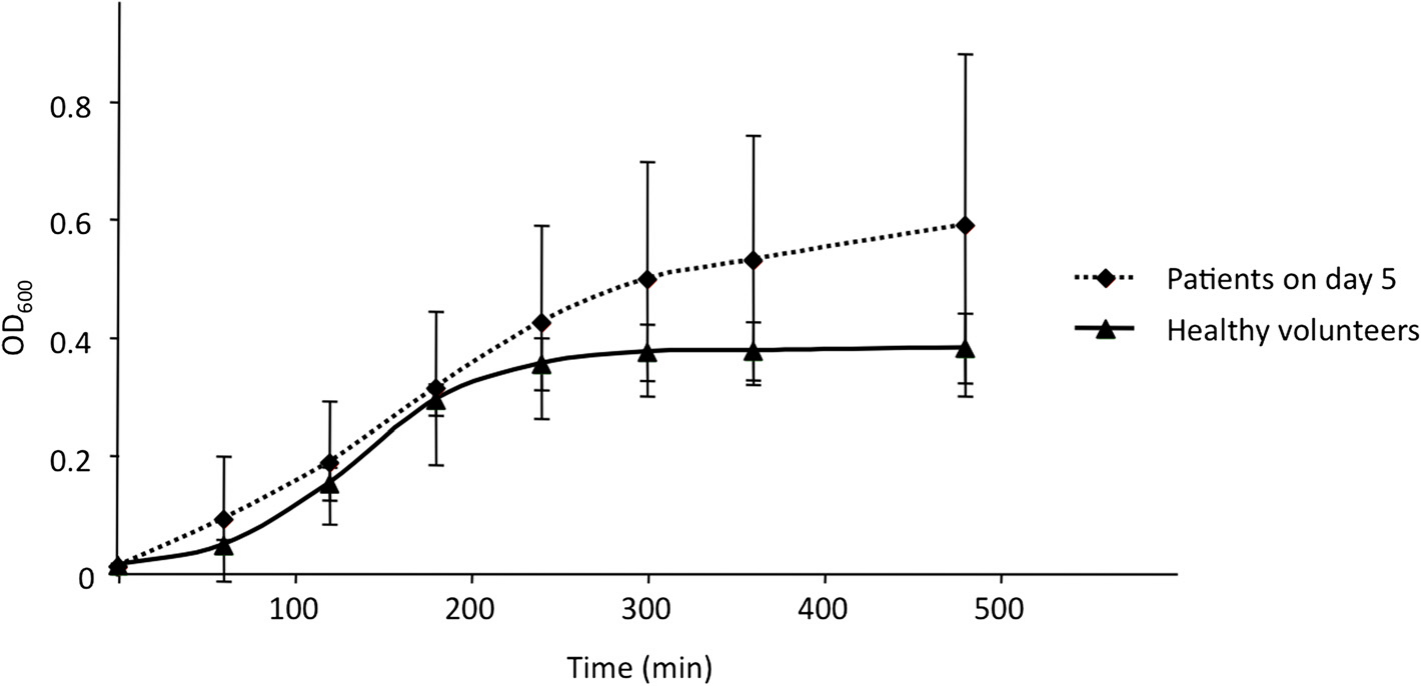
**Comparison of the growth of *E. coli *CFT073 in urine from trauma patients not receiving antibiotic collected on day 5 (n = 13) (dashed line), and from healthy volunteers (n = 12) (complete line) (*p *= 0.0108).**

Growth of *E. coli* CFT073 was evaluated for the 13 urine samples from patients without bacteriuria and without antibiotic on day 5. The growth in these 13 samples was statistically greater than in HV urine (*p* = 0.0108) (Figure 4). Results similar to the *E. coli* CFT073 strain were found for *E. coli* R1B8J20 grown in the 8 tested samples from the 13 patients without antibiotics and compared to HV urine growth (*p* = 0.0478). One of the 2 patients with antibiotics on day 5 had received gentamicin; as both strains of *E. coli* were susceptible to this antibiotic, only the sample from the patient who did not received gentamicin was evaluated. The bacterial growth was higher than in HV urine (data not shown).

To test the role of iron in the bacterial growth, we carried out inhibition experiments using an iron chelator in urine from 3 patients who did not received antibiotics at day 1. Addition of desferrioxamine (0.1 mM) in trauma urine suppressed growth, with a decrease in the number of bacteria at the stationary phase of around 40% (± 20%).

The addition of norepinephrine (1 μM, 10 μM and 50 μM) to HV urine does not change *E. coli* CFT073 strain proliferation in our experimental conditions (data not shown).

### Urinary biochemical assays

On day 1, the levels of urea and creatinine, and the urinary osmolarity, were significantly lower in the urine of trauma patients (TP urine) than in HV urine (Table 3). Moreover, all of the patients had a glycosuria, and 80% of them had subnormal levels of urinary iron (Table 4). Seventy-seven per cent of the patients had received a norepinephrine infusion on day 1. The urinary norepinephrine concentration was above normal values in 75% of patients receiving norepinephrine on day 1 and in 40% of patients who had not received catecholamines (Table 4).

**Table 3:** Comparison of biochemical characteristics between urine from trauma patients on day 1 (D1) and from healthy volunteers (HV)

**Variables**	**Values D1**	**Values HV**	**HV-D1 mean or median differences [CI95%]**
Urea (mmol/L)	134 ± 76	296 ± 59	162 [104; 220]
Creatinine (μmol/L)	6.31 ± 4.04	10.55 ± 3.29	4.25 [1.14; 7.36]
Sodium (mmol/L)	80 ± 44	104 ± 43	29.28 [−7.09; 65.65]
Potassium (mmol/L)	44 ± 28	51 ± 20	9.83 [−11.44; 31.11]
Proteinuria (g/L)	0.57 ± 0.67	0.017 ± 0.025	−0.55 [ −1.11; 0.01]
pH	6.08 ± 1.02	6.7 ± 0.32	0.60 [−0.04; 1.25]
Osmolality (mosmol/L)	273 ±129	515 ± 92	241.76 [160.66; 322.86]
Nitrates (nmol/mg of protein)	34 ± 57	63 ± 45	31.25 [10.55; 51.95] ^*a*^

*D1* on day 1.

^*a*^ Medians difference and Hodges-Lehmann 95% Confidence Interval.

**Table 4:** Urinary concentrations of catecholamines, iron and glucose for patients at day 1 and day 5 and percentages of patients with concentrations above the normal

**Variables**	**D1**	**D5**
	**Values (means ± SD); % above the normal [CI 95%]**	**Values (means ± SD); % above the normal [CI95%]**
Dopamine > 630 (μg/g creatinine)	158 ± 53 ; 0.0 [0.00 ; 28.49]	154 ± 74 ; 0.0 [0.00; 28.49]
Epinephrine > 16 (μg/g creatinine)	15.3 ± 11.7; 50.0 [21.09; 78.91]	14.2 ± 10 ; 36.4 [10.93; 69.21]
Norepinephrine > 77 (μg/g creatinine)	219 ± 223 ; 69.2 [38.57; 90.91]	153 ± 195 ; 45.5 [16.75; 76.62]
Iron (μmol/L) > 0	0.65 ±1.02 ; 81.3 [54.35; 95.95]	0.24 ± 0.55 ; 41.2 [18.44; 67.08]
Glucose (g/L) > 0	1.45 ± 1.14 ; 100.0 [79.41; 100.00]	0.78 ± 0.61 ; 100.0 [80.49; 100.00]

*D1* on day 1, *D5* on day 5.

On day 5, glycosuria was present in all patients, and proteinuria was higher in TP urine than in HV urine. There was no difference in urinary osmolarity, and urinary iron and urea concentrations between TP and HV urines (Tables 4 and 5). Less than 45% of the patients had a urinary concentration of norepinephrine above normal values (Table 4), and three fourth of them still received catecholamines.

**Table 5:** Comparison of biochemical characteristics between urine from trauma patients on day 5 and from healthy volunteers

**Variables**	**Values D5**	**Values HV**	**HV-D5 mean or median differences [CI95%]**
Urea (mmol/L)	225 ± 149	296 ± 59	83 [14; 179] ^*a*^
Creatinine (μmol/L)	7.15 ± 4.45	10.55 ± 3.29	3.41 [0.02; 6.79]
Sodium (mmol/L)	95 ± 46	104 ± 43	13.30 [−24.42; 51.02]
Potassium (mmol/L)	36 ± 24	51 ± 20	20.45 [2.60; 38.30] ^*a*^
Proteinuria (g/L)	0.34 ± 0.31	0.017 ± 0.025	−0.32 [−0.55; -0.09]
pH	7.35 ± 0.83	6.7 ± 0.32	−0.67 [−1.30; -0.04]
Osmolality (mosmol/L)	418 ± 180	515 ± 92	99.75 [−29.00; 228.50] ^*a*^
Nitrates (nmol/mg of protein)	56 ± 46	63 ± 45	9.83 [−32.87 ; 52.52]

Values are expressed as means ± SD, *D5 *on day 5, *HV* healthy volunteers.

^*a *^Medians difference and Hodges-Lehmann 95% Confidence Interval.

Analysis of amino acids revealed an increase of urinary excretion of glutamic acid, valine, methionine, leucine, tyrosine, histidine and phenylalanine during the first 24 hours after trauma (Table 6). By the fifth day post-trauma, the excretion of almost all amino acids had significantly increased (Table 7).

**Table 6:** Comparison of urinary amino acid concentrations between trauma patients on day 1 and healthy volunteers

**Amino acids**	**D1**	**HV**	**HV-D1 mean or median differences [CI95%]**
**(μmol/L)**	**Mean ± SD**	**Mean ± SD**	
Taurine	738 ± 551	944 ± 597	205.98 [-390.38; 802.35]
Aspartic acid	14 ± 23	8 ± 2	-6.43 [-22.53; 9.66]
Threonine ^*a*^	339 ± 519	105 ± 23	-233.38 [-601.06; 134.31]
Serine ^*a*^	525 ± 596	242 ± 42	-283.04 [-686.74; 120.66]
Asparagine ^*a*^	258 ± 329	72 ± 17	-186.31 [-419.08; 46.47]
Glutamic acid	83 ± 133	10 ± 7	-34.29 [-56.20; -12.37] ^*b*^
Glutamine ^*a*^	650 ± 608	326 ± 76	-323.98 [-757.79; 109.83]
Glycine ^*a*^	1991 ± 2396	879 ± 211	-1111.23 [-2813.86; 591.41]
Alanine ^*a*^	407 ± 273	240 ± 56	-167.22 [-365.07; 30.64]
Citrulline	74 ± 149	15 ± 5	-59.03 [-164.57; 46.51]
Valine	96 ± 69	36 ± 9	-60.71 [-109.93; -11.48]
Cystine	205 ± 337	35 ± 9	-73.56 [-145.20; -1.92] ^*b*^
Methionine ^*a*^	15 ± 11	5 ± 6	-10.23 [-19.00; -1.46]
Isoleucine	26 ± 13	17 ± 4	-9.02 [-19.04; 1.00]
Leucine	61 ± 49	18 ± 3	-43.30 [-77.81; -8.79]
Tyrosine	213 ± 167	76 ± 30	-137.20 [-257.38; -17.03]
Phenylalanine	433 ± 360	55 ± 10	-378.06 [-632.89; -123.23]
Ornithine	135 ± 339	7 ± 2	-13.81 [-26.96; -0.67] ^*b*^
Histidine	1469 ± 1347	543 ± 159	-768.69 [-1447.56; -89.82] ^*b*^
Lysine	2000 ± 5164	104	-1896.22 [-5550.61; 1758.18]
Arginine	71 ±147	11 ± 95	-85.12 [-238.12; 67.88]

*D1 *on day 1, *HV *healthy volunteers.

^*a *^Amino acids known to be particularly important during growth in urine for *E. coli *(personal data and [11,17,18,24].

^*b *^Medians difference and Hodges-Lehmann 95% Confidence Interval.

**Table 7:** Comparison of urinary amino acid concentrations between polytrauma patients on day 5 and healthy volunteers

**Amino acids**	**D5**	**HV**	**HV-D5 mean or median differences [CI95%]**
**(μmol/lL)**	**Mean ± SD**	**Mean ± SD**	
Taurine	324 ± 301	944 ± 597	619.36 [87.15; 1151.57]
Aspartic acid	12 ± 15	8 ± 2	-4.45 [-14.94; 6.03]
Threonine ^*a*^	778 ± 382	105 ± 23	-672.79 [-944.09; -401.49]
Serine ^*a*^	841 ± 429	242 ± 42	-598.42 [-903.86; -292.97]
Asparagine ^*a*^	459 ± 240	72 ± 17	-387.51 [-557.85; -217.18]
Glutamic acid	57 ± 49	10 ± 7	-46.82 [-81.78; -11.85]
Glutamine ^*a*^	864 ± 365	326 ± 76	-538.03 [-803.53; -272.53]
Glycine ^*a*^	2049 ± 985	879 ± 211	-1170.10 [-1888.18; -452.03]
Alanine ^*a*^	390 ± 202	240 ± 56	-123.41 [-268.06; 21.25] ^*b*^
Citrulline	73 ± 57	15 ± 5	-58.67 [-98.89; -18.44]
Valine	79 ± 46	36 ± 9	-43.29 [-76.66; -9.91]
Cystine	225 ± 209	35 ± 9	-189.76 [-337.97; -41.56]
Methionine ^*a*^	9 ± 8	5 ± 6	-4.23 [-11.74; 3.28]
Isoleucine	22 ± 16	17 ± 4	-5.13 [-17.04; 6.78]
Leucine	52 ± 32	18 ± 3	-34.34 [-57.07; -11.61]
Tyrosine	222 ± 134	76 ± 30	-146.75 [-244.35; -49.16]
Phenylalanine	258 ± 187	55 ± 10	-202.73 [-334.94; -70.51]
Ornithine	99 ± 132	7 ± 2	-93.38 [-173.72; -13.05] ^*b*^
Histidine	1267 ± 575	543 ± 159	-724.23 [-1151.22; -297.24]
Lysine	1542 ± 1993	104	-1437.41 [-2849.66; -25.15]
Arginine	54 ± 64	11 ± 95	29.48 [-86.49; 145.45]

*D5 *on day 5, *HV *healthy volunteers.

^*a *^Amino acids known to be particularly important during growth in urine for *E. coli* (personal data and [11,17,18,24].

^*b *^Medians difference and Hodges-Lehmann 95% Confidence Interval.

## Discussion

UTIs are one of the most important hospital-acquired infections in ICUs and are responsible for a large proportion of antibiotics prescribed. Few of the known risk factors are preventable, making it important to improve our understanding of the underlying causes of UTIs. In our study we have demonstrated that urine of severe trauma patients favours the growth of *E. coli*, which is the most frequently isolated species in UTIs [7,25]. This effect is present during the first 24 hours, and remains at least until the fifth day after occurrence of trauma.

The changes in urinary composition induced not only by shock and trauma, but also by the urinary catheter could facilitate bacterial growth of both susceptible and resistant strains. Comparing the urine from trauma patients with that from healthy volunteers highlights a significant increase in glycosuria, urinary amino acids, urinary iron and norepinephrine concentrations, and a significant decrease in urinary urea concentrations and osmotic pressure. These could, together, play a role in the difference seen in bacterial growth. When norepinephrine was added to HV urine this alone did not facilitate bacterial growth.

Critically ill patients are often characterized by insulin resistance associated with impaired glucose tolerance and hyperglycemia. This stress induced-hyperglycemia leads to glycosuria, which is known to support bacterial growth in urine [26,27].

The changes in urinary amino acids concentration that we have shown are also well known to influence growth of *E. coli* in urine [9,11,28]. Bacterial growth in urine is dependent on the availability of nitrogen. Some amino acids, such as serine or glutamic acid, increase *E. coli* growth [29,30] (personal data). In critically ill patients, anabolic resistance and increased energy requirements lead to proteolysis, with a corresponding increase in the efflux of amino acids. Systemic inflammation alters amino acid transport through muscle cells. The increase in plasma amino acids levels could lead to a rise in urinary excretion [31-33]. In agreement with the work of Freund et al., we found that sulphur- containing amino acid (methionine), aromatic amino acids (phenylalanine and tyrosine) and branched chain amino acids (leucine and valine) are excreted early [34]. Then, at the fifth day we showed an increase in the levels of excretion of the majority of amino acids as the result of catabolism, as well as renal damage in some cases. The urine iron level in healthy individuals is usually too low to support bacterial growth [9]. The occurrence of phenomena such as rhabdomyolysis, blood transfusion or urinary tract trauma and also urinary catheter insertion could explain the higher iron levels. In our study, more than 80% of trauma patients had rhabdomyolysis, which leads to leakage of muscle proteins containing ferrous iron [35]. Half of the patients were transfused during the first five days of their hospitalization, usually on the first day, with a mean of 3.4 units per patient. Blood transfusion can lead to intravascular haemolysis and haemoglobinuria. The role of iron in stimulating bacterial growth is confirmed by our data showing growth inhibition after addition of desferrioxamine, an iron chelator.

Some pathophysiological changes occurring in trauma patients could lead to increased *E. coli* growth in urine by a non-nutritional effect. There is a lower urea concentration in urine from trauma patients on day 1, in comparison with HV urine, which could be explained by the decrease in protein input and hypovolemia. Urea has an antibacterial effect, which is independent of osmotic pressure, but can also influence bacterial proliferation through osmotic pressure [9,36].

The potential role of urinary catecholamines could be explained by their inhibitory effect on bactericidal activity of leukocytes [37] and by their own effect on bacterial multiplication [23,38,39]. Several studies have reported that catecholamines stimulate bacterial proliferation. However, these studies focused on gut flora and did not explore bacterial growth in the urinary tract. With low bacterial inoculum and during prolonged bacterial growth, Freestone et al. have shown a positive effect of norepinephrine on bacterial growth even at 1 μM, the mean concentration of norepinephrine in the TP urine [23]. We could not demonstrate under our experimental conditions a positive and independent effect of norepinephrine on bacterial growth, suggesting that the effect of catecholamine alone is weak and requires the presence of iron to stimulate bacterial multiplication.

Finally, in 5 cases bacteria were present in the urine; they were in two cases *P. mirabilis*. These findings highlight the predisposition of trauma patients to develop asymptomatic bacteriuria.

Strengths of our study include i) its originality with a new concept which could contribute to high sensitivity of critically ill patients to infections; ii) the exploration of nutritional and non-nutritional way to facilitate bacterial growth; ii) the comparison between patients and healthy volunteers. However, our study has several limitations The list of metabolites that we studied is not exhaustive; for example, we did not explore interleukin (IL) urinary excretion, even though IL-1 is known to be excreted during acute kidney injury due to shock and is known to enhance *E. coli* growth [24,40]. Eighteen patients received antibiotics at one stage of the study, making non interpretable some urine samples. The fact that the healthy volunteers did not have an indwelling bladder catheter may be a confounding factor, because the urinary catheter could change the urinary sediment or cause cellular damage. If further investigations are required to discriminate the potential role played by the urinary catheter, the trauma and shock remain the main cause of the increase in glycosuria, in amino acid excretion and in change in urea concentration.

## Conclusions

This study demonstrates that urine of trauma patients supports *E. coli* growth better than urine from healthy volunteers. This effect is present from the first 24 hours and remains at least until the fifth day after trauma. The effect is observed with antibiotic-resistant strains for patients who have received antibiotics, highlighting the impact of antibiotics on selection of resistant strains. The changes in urinary glucose, amino acid and iron concentrations, and urinary urea and osmotic pressure could all play a part in the observed increase in *E. coli* proliferation. This increase in the growth ability of *E. coli* strains in urine of trauma patients could be one of the elements facilitating UTIs in these patients. A better understanding of the occurrence of bacteriuria is needed to define interventions that could decrease the incidence of UTIs in the context of the intensive care unit.

## Competing interests

The authors have no competing interests.

## Authors’ contributions

CA contributed to the conception and design of the study, bacterial experiments, results analysis and wrote the article. OH contributed to the conception and design of the study, did the statistical analysis and reviewed the manuscript. SR supervised patients’ enrolment, the acquisition of patient data, participated in the results analysis and reviewed the manuscript. DB carried out biochemical dosages and reviewed the manuscript. EP carried out hormonology experiments and their analysis, and reviewed the manuscript. PEL participated to patients’ enrolment and reviewed the manuscript. OB contributed to conception and design of the study, bacterial experiments and reviewed the manuscript. EV participated in the statistical analysis and review the manuscript. ED supported the study conception and participated in the manuscript writing. JD contributed to conception and design of the study, to the result interpretation and to the writing of the manuscript. All authors read and approved the final manuscript.

## Authors’ information

CA was funded by an INSERM fellowship.

## Pre-publication history

The pre-publication history for this paper can be accessed here:

http://www.biomedcentral.com/1471-2334/12/330/prepub
